# Thermotolerance of Broiler Chicks Ingesting Dietary Betaine and/or Creatine

**DOI:** 10.3390/ani9100742

**Published:** 2019-09-29

**Authors:** Hosam Al-Tamimi, Kamel Mahmoud, Amani Al-Dawood, Basheer Nusairat, Hussam Bani Khalaf

**Affiliations:** 1 Department of Animal Production, Faculty of Agriculture, Jordan University of Science and Technology, Irbid 22110, Jordan; hjaltamimi@just.edu.jo (H.A.-T.); kmahmoud@just.edu.jo (K.M.); bmnusairat@just.edu.jo (B.N.); hussambanykhalaf@yahoo.com (H.B.K.); 2 Department of Applied Biology, College of Sciences, Tafila Technical University, Tafila 66110, Jordan

**Keywords:** broilers, thermophysiological, heat stress, dietary supplement, welfare

## Abstract

**Simple Summary:**

Improving broiler performance is still an essential task in animal production, especially under certain environmental challenge conditions. Heat stress is among the first and crucial limiting factors of the development of poultry production in warm regions. Betaine (B) and creatine (C) are seemingly two promising additives that showed enhancement in water metabolism in several animal species. Despite the clear beneficial effect of B and/or C on water metabolism, limited information is available about their use and effect on performance and thermophysiological responses of broiler chicks exposed to heat stress challenge. In view of the above consideration and in order to improve the productivity of broiler chickens, the present study was designed to examine the potential alteration in water metabolism in broiler chicks treated with B and/or C under thermoregulation challenge, which may contribute to the ongoing efforts to improve broiler chicken production. Under the current study conditions, the results demonstrated that performance and carcass parameters measured were not affected by B and/or C supplementation by the end of the rearing period. In light of the improved thermoregulatory performance and water balance indicators, it seems that B and/or C have successfully improved water holding capacity and therefore helped in enhancing thermo-tolerance.

**Abstract:**

The present study aimed to assess the effect of dietary betaine (B) and/or creatine (C) on performance and thermoregulatory responses of broiler chicks. Indian River broiler chicks, fitted with compact thermosensors, were reared to market age (five weeks). The chicks were randomly distributed into four treatment groups, in a 2 × 2 factorial arrangement of treatments—basal control diet (Control group: CONT; B−/C−); 1 g betaine/kg feed (Betaine group: BETA; B+/C−), 1.2 g creatine monohydrate/kg feed (Creatine group: CRET; B−/C+), and combination (Betaine and Creatine group: COMB; B+/C+) of both supplements. At 31 days of age, 20 chicks from each group were exposed to acute heat stress (A-HS) for 3 h (34.45 ± 0.20 °C), and hemogramic profiles were screened before and after. Performance parameters (feed intake, body weight gain, and feed conversion ratio) were reported on a weekly basis, and carcass meat quality was evaluated at the end of experiment. Redness of breast was higher due to B and C treatments separately than the CONT group (B by C interaction; *p* < 0.05). Compared to the CONT, dietary supplements alleviated hyperthermia responses, with B alone being more efficient than C or COMB treatments. The mitigation of hyperthermia is likely mediated by enhancement of water balance indicators. Although not efficient in improving growth performance, dietary B and/or C are efficient in improving thermophysiological performance and survival of finishing broiler chicks under A-HS.

## 1. Introduction

The poultry industry is recognized as the most popular emerging industry in the world [1,2,3]. Chicken meat represents a good and cheap protein source compared to red meat. In addition, short productive lifespan, egg production, dietary restriction absence, and worldwide distribution are all favored the use of poultry products as a major source of animal protein [4,5]. The Jordanian broiler production industry has been developed very fast in the last decades and has become one of the most important sectors in the animal production industry that contributes to employing manpower, and its revenue is the main source of income for agricultural holders [6].

Improving broiler performance is still an essential task in animal production, especially under certain environmental challenge conditions. The use of dietary supplements has been long attempted to achieve this goal [7,8,9,10]. Betaine (B) and creatine (C) are seemingly two promising additives that showed enhancement in water metabolism in several animal species [11]. Betaine is an osmolyte that contains glycine betaine and trimethylglycine and is naturally found in certain plant species as a nontoxic amino acid derivative [12]. Betaine has numerous functions in animals, including aiding in cell volume regulation under osmotic stress, by activity of betaine-homocysteine methyltransferase betaine, which is capable of donating a methyl group to homocysteine and hence helps in regeneration of methionine, and the transmethylation cycle reaction can therefore reduce methionine requirements [13]. Betaine usage in poultry feeding improves both feed conversion ratio (FCR) and final body weight (BW), especially when homocysteine levels are limiting [7].

Creatine “*N*-[aminoiminomethyl]-*N*-methyl glycine” or “methyl guanidine acetic acid” is a guanidine compound synthesized by the liver, kidney, pancreas, and brain, being a precursor of amino acids such as arginine, glycine, and methionine. In addition, it is involved in the regulation of cellular energy demand. Creatine represents one of the most important nitrogen-containing compounds in protein and energy metabolism. It is an organic compound that is directly involved in the muscle energy buffering system [14]. Furthermore, in humans, it has been found to elevate total muscle creatine content and muscle mass, as it aids in the maintenance of primary myotubes [15]. Other studies also showed advantageous effects of C supplementation on ATP replenishment, intramuscular contents of phosphocreatine, and glycogen in both white and red muscles. Also, C reportedly reduces pH 3–4 h postmortem and increases both lightness unit and water holding capacity in broiler chicks. Likewise, C was also found to delay the onset of fatigue in active muscles by enhancing phosphorylation to improve co-administration with glucose, with C enhancing insulin sensitivity and the proteogenic pathway in muscle. 

Animals undergo various kinds of stressors, i.e., physical, nutritional, chemical, psychological, and thermal stressors. Among all, thermal/heat stress (HS) is the most concerning issue in the ever-changing climatic scenario, and it is one of the most important stressors, especially in the tropical, subtropical, arid, and semiarid regions of the world [16,17,18]. Research assessment has concluded that measures to evaluate the negative welfare in poultry linked to stress and stressful conditions that adversely affect behavior, production, and physiology [19]. Traditionally, the most important criteria for evaluating the performance of the broiler strains have been the growth rate, FCR, and carcass composition [20]. Despite the clear beneficial effect of B and/or C on water metabolism, limited information is available about their use and combinational effect on performance and thermophysiological responses of broiler chicks exposed to HS. In view of the above consideration and in order to improve the productivity of broiler chickens, the present study has been designed to examine the potential alteration in water metabolism in broiler chicks treated with B and/or C under thermoregulation challenge and thus contributes to the ongoing efforts to improve broiler chicken production in Jordan.

## 2. Materials and Methods

### 2.1. Location, Birds, and Experimental Procedure

The study consisted of two parts—a performance study conducted at the poultry farm (Agriculture Center of Research and Production) and a thermoregulation study carried out at the Animal House (Jordan University of Science and Technology, Irbid, Jordan). In the performance trial, a total of 440 one-day-old straight run Delaware broiler chicks were obtained from a local commercial hatchery. The Delaware, once named Indian River, was developed in the United States. The fowl is a cross between a Plymouth Rock and a New Hampshire. The Delaware is a white bird with black barred plumage near the neck area. The tail feathers are also black barred. The fowl has a single red comb and red wattle and earlobes. The shanks are typically yellow, and the skin is white. 

All chicks received a mixed vaccine (infection bronchitis + New Castle disease) right after hatching. The birds were randomly assigned to four dietary treatments, five replicates each: (1) basal control diet (Control group: CONT; B−/C−) (Table 1); (2) a basal diet containing 1 g betaine/kg feed (Betaine group: BETA; B+/C−); (3) basal diet containing 1.2 g creatine monohydrate/kg feed (Creatine group: CRET; B−/C+); and (4), or a basal diet containing B and C (Betaine and Creatine group: COMB; B+/C+) at the previously mentioned levels. At one day of age, birds were randomly allocated to 20 floor pens (2 × 1 m) with wood shavings used as bedding (22 birds/replicate). The floor pens were located in an open-sided poultry house. Each pen was prepared with one automatic bell drinker and one tube feeder. A replicate was therefore the experimental unit in this experiment. All birds were reared and grown to market age (35 days) under standard commercial conditions. All procedures were pre-approved by the Institutional Animal Care and Use Committee at the Jordan University of Science and Technology (number 338/2015). On day 14, 180 randomly pre-assigned chicks underwent a minor invasive procedure to implant thermal chips (LifeChip^®^, Desetron Fearing, Dallas, TX, USA).

The skin under the left wing was first rubbed with medical grade alcohol to disinfect the implantation site. Then, the chip was inserted sub-dermally to a caudal distance of 4 cm in a 45° angle (to the vertical standing plane) with the bevel of the injection needle pointing out. Then, the site was disinfected with a gentamycin spray. Nine birds from each pen were ID-marked using a color spray (Gentian Violet) at distinct body locations for individual core body temperature (Tcore) monitoring. No signs of irritation or inflammation were noticed on any of the implanted birds throughout the trial. On day 29, 80 (thermal chip–implanted; will be described below) birds were transported to the Animal House (20 birds/treatment) and were allowed a 48 h adaptation period prior to initiation of the acute heat stress (A-HS) study phase on day 31. Also, to rule out the potential interference of this procedure, performance parameters of the implanted birds were compared to un-implanted counterparts throughout the study and were found to be homogeneously indifferent. Between 08:30 and 09:30, on days 26, 27, 28, 30, and 34, Tcore readings from all 180 birds were collected using handheld scanners (DTR5-60 and Portable Global Pocket Reader^®^, Desetron Fearing, Dallas, TX, USA). In parallel, ambient temperature and RH (%) were monitored on an hourly basis for the duration of the trial, using miniature thermologgers (iButton, 1922L, Embedded Data Systems, Lawrenceburg, KY, USA), distributed at 10 different locations within the poultry farm at bird level. The light cycle was kept constant throughout the whole study duration (23 h light, and 1 h of darkness, from 05:00 to 06:00).

### 2.2. Growth Performance Study

Two diets were used, a starter from day 1 to 14, and a grower from day 15 to 35, according to the recommended commercial guideline. Feed addition and remaining feed weigh back were performed weekly to calculate feed intake (FI). Chicks (in tens/pen) were weighed at the day of arrival, and the average one-day-old weight (g) was calculated (initial BW). Birds in each replicate were weighed as a group weekly and body weight gain (BWG) and FCR were calculated. 

### 2.3. Slaughter Procedure and Carcass Traits Measurements

At the end of the rearing period, water and feed were removed at night (8 h before slaughtering). In the next morning, four birds/replicate/treatment were randomly chosen and weighed before slaughter. All birds were exsanguinated by manually severing both the carotid artery and at least one jugular vein with a knife, birds were allowed to bleed for 120 s, and then the head and shanks were removed. Thereafter, birds were scalded at 60 °C for 120 s followed by defeathering. All carcasses were then eviscerated, washed, and chilled. Breast cuts were excised from carcasses by cutting them into forequarters and leg quarters as described by Hudspeth et al. [21]. The wings were separated from the carcass at the shoulder, leaving as much meat as possible on the breast. The breast muscles were separated from the back at the shoulder by severing the humeral–scapular joint and pulling it downward to strip the meat from the breast. The breast, drumstick, wings, backbone, and neck weights were recorded. The percentages of the individual parts are based upon the combined weight of the parts obtained after cutting. Internal muscle pH and temperature were directly measured through an incision made by a knife in the right pectoralis major muscle using a portable pH meter (pH spear, large screen, waterproof pH/temperature tester, double injection, model 35634–40, Eurotech instruments, Kuala Lumpur, Malaysia), and a digital thermometer (Electro-term, model TM 99A, Cooper Instrument Corporation, Middlefield, CT, USA). Whole breast from each carcass was then individually placed in labelled sealed polyethylene plate, wrapped in wax paper, and placed in a freezer at −32 °C.

#### 2.3.1. Meat Quality Measurements

Frozen breasts were thawed overnight in a refrigerator at 4 °C. Then, the whole breast samples were cut up and deboned. Care was taken to ensure that all fillets were removed in the same manner so that the meat quality variables would not be affected by the deboning procedure. Left and right pectoralis major muscles without skin were obtained from breast fillets. For meat quality analysis, left pectoralis muscle was used for measurements of cooking loss and tenderness, whereas the right pectoralis major muscle was used for color, pH, water holding capacity, water activity and chemical composition measurements. Left pectoralis muscle was weighed (initial weight), then placed in labelled polythene bags. The bags were placed in thermostatically controlled water bath and cooked for 25 min at 85 °C to achieve the maximum internal temperature of 80 °C. After cooking, the bags were temperate at room temperature before opening to drain the liquid, and then the cooked sample was dried with paper towel to remove excess surface moisture and re-weighed. Cooking loss was reported as the weight lost during cooking divided by fresh sample weight and expressed as a percentage [22]. In order to investigate the tenderness, within 3 h of cooking, the dried samples from the cooked meat were cut to obtain five cores (20 × 13 × 13 mm) with similar size parallel to a line beginning at the humoral insertion and ending at the point adjacent to the keel and include the complete depth of each cooked muscle sample. Each core was sheared perpendicular to the longitudinal orientation of the muscle fiber using a Warner–Bratzler shear blade with the triangular slot cutting edge mounted on Salter model 235 (Warner-Bratzler meat shear, G–R manufacturing Co. 1317 Collins LN, Manhattan, KS, USA) to determine the peak force (kg) needed to shear the samples. Shear force was determined as the average of the five replicates from each pectoralis major muscle sample [22].

The pH values were determined in duplicate samples using the iodoacetate method as described by Sams and Janky [23]. One to 1.5 g of raw right muscles were put into plastic test tube containing 10 mL of neutralized 5 mM iodoacetate reagent and 150 mM KCl, and homogenized using homogenizer (Ultra–Turrax T8, IKA Labortechnik, Janke & Kunkal GmbH & Co., Staufen, Germany). The pH values of the solutions were recorded using a pH meter (pH Spear, Large screen, waterproof pH/temperature Tester, double injection, model 35634–40, Eurotech instruments, Kuala Lumpur, Malaysia). Instrumental color measurements of raw right pectoralis muscles were measured 24 h post thawing using colorimeter (12MM Aperture U 59730–30, Cole-Parameter International, Accuracy Micro sensors, Inc. Pittsford, NY, USA), calibrated throughout the study using a standard white ceramic reference (CIE L* = 97.91, a* = −0.68, b* = 2.45). Samples were placed on tray, covered with wax paper (to avoid surface drying) and left in the refrigerator for about 2 h to allow enough contacts with atmospheric oxygen to turn them to the normal color. Random readings were taken at three different locations on the muscle surface that has been adjacent to the skin, at an area free of any noticeable color defects. Values were averaged, and color for each sample was expressed in terms of CIE LAB (commission International IE Clairage) brightness (L*), redness (a*), and yellowness (b*). The water-binding properties of the pectoralis major muscle were estimated by measuring the amount of water released from the muscle protein by the application of force (expressible juice) and by measuring the ability of muscle protein to retain water present in excess and under the influence of internal force (water holding capacity). Water holding capacity was measured using the method described by Horcada et al. [24] using samples of approximately 5 g raw meat (initial weight). Each meat sample was cut into small pieces, and each piece was covered with two filter papers (qualitative, 185 mm ф circles, fine crystalline retention, Whatmam International Ltd., Maidstone, England) and two thin plates of quartz material were then pressed with a weight of 2500 g for 5 min. The meat samples were then removed from the filter paper, and their weight was recorded (final weight). Water-holding capacity was reported as the weight lost during sample pressing divided by initial sample weight and expressed as a percentage.

#### 2.3.2. Blood Serum Measurements

At 31 days of age, blood samples were taken from eight chicks within each treatment (two chick/replicate) after and/or before exposure to HS. Serum was obtained by centrifuging blood at 4000 round/min for 10 min. Collected serum was immediately stored at −20 °C for blood serum measurements. White blood cell, red blood cell, hemoglobin, hematocrit, mean corpuscular volume (MCV), mean corpuscular hemoglobin (MCH), mean corpuscular hemoglobin concentration (MCHC), thrombocyte, heterocyst, basophils, lymphocytes, monocytes, and heterocyst/lymphocyte ratio were measured.

### 2.4. Heat Stress

The procedures were reviewed and approved by the Animal Care and Use Committee (number 338/2015) at the Jordan University of Science and Technology, and the trial was conducted at the on-campus Animal House Facility. In a climatically controlled room, thermoneutrality (TN; Ta = 25.77 ± 0.28 °C; RH = 32.28 ± 0.40%) was maintained throughout the first 48 h adaptation phase prior to onset of the A–HS bout (Ta = 34.45 ± 0.20 °C; RH = 37.60 ± 0.28%). All 80 chicks were group-kept in four cages, with each being equipped with individual gravity feeders and a water nipple line. The room temperature and RH (%) were monitored with data loggers for the whole experiment duration. Starting at 11:00 h of day 31, the A-HS bout was initiated by elevating ambient temperature from 25.77 °C to 34.45 °C for a period of 150 min. Then, TN was resumed again, and Tcore values were scanned and recorded at 0, 30, 90, 150, 270, and 330 min post A-HS.

### 2.5. Statistical Analysis

Performance data (BW, FCR, FI, and BWG) were initially analyzed by analyses of variance (ANOVA) using General Linear Models (GLM) procedure of SAS (SAS Institute, Inc., Cary, NC, USA) [25] as split plot in time. However, due to absence of time by (any) treatment interaction (with betaine nor creatine), data were analyzed again for each week (using the replicate within treatment as the error term). Likewise, meat quality data were analyzed similarly. On the other hand, body temperature data were analyzed using ANOVA using MIXED Procedures of SAS (as extensively explained by Littell et al. [26]) as split-plot in time for repeated measures, in which the chick within treatment was used as the error term. In this latter procedure, all three possible covariate structures (compound symmetry, autoregressive and unstructured) were initially tested, and we finally found the autoregressive structure [AR(1)] to be most appropriate and used it. Finally, blood data were analyzed using the latter procedures. Data were finally presented as least square means ± 1 standard error (of the means), and significance was declared when *p* value was ≤ 0.05.

## 3. Results

### 3.1. Broiler Growth Performance

Broiler performance parameters (BW, FCR, FI, and BWG) are presented in Table 2. Time (age; week) was primarily responsible for significant (*p* < 0.05) changes on all parameters, without any interaction found with the either treatments (*p* > 0.05). At age of two weeks, a significant improvement in FCR was triggered by creatine (in both CRET and COMB groups; *p* < 0.01), but this effect vanished later on. This C effect was parallel to significantly (*p* = 0.03) greater BWG at the same period. No other growth performance-related parameters were observed by either treatment or by their combination.

### 3.2. Breast Meat Quality and Color Values

The tested meat quality variables—carcass dressing (%), pH, breast meat cooking loss (%), water holding capacity (WHC%), shear force (SF) kg/cm2, and color values (lightness, redness, and yellowness)—are presented in Table 3. Betaine and/or C supplementation did not significantly (*p* > 0.05) affect any of the examined parameters. 

Breast meat color values (lightness and yellowness) displayed no differences due to inclusion of B and/or C in the diet. A creatine by B interaction (*p* < 0.05) was found with reference to meat redness, being lower in both CRET and BETA than in the CONT or when combined (COMB).

### 3.3. Thermoregulation and Hemogramic Profile

**Figure 1:**
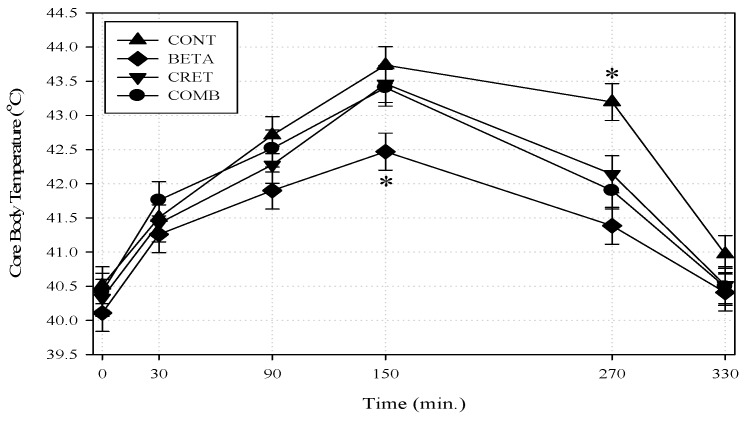
Core body temperatures (°C) of acutely heat-challenged broiler chicks treated with dietary betaine (B; 1 g/kg diet), creatine (C; 1.2 g/kg diet) or B + C combination (Betaine and Creatine group: COMB). Broiler chicks (age 32 days; *n* = 20) were exposed to 150 min of heat stress (Ta = 34.45 ± 0.20 °C, RH% = 37.60 ± 0.28%) after which thermoneutrality was resumed (Ta = 25.77 ± 0.28 °C, RH% = 32.28 ± 0.40%). All 80 chicks were brought from floor open system farming 48 h prior to the heat challenge test for new–housing adaptation. The control group (CONT) contained neither added B nor C. First and second asterisks indicate B by time, and B by C by time interactions; respectively (*p* < 0.05). BETA: Betaine group, CRET: Creatine group.

The A–HS bout lasted for 150 min (aver. Ta = 34.45 ± 0.20 °C; RH = 37.60 ± 0.28%), after which thermoneutrality was restored (Ta = 25.77 ± 0.28 °C; RH = 32.28 ± 0.40%). All Tcore values arose significantly (*p* < 0.01) with time during the first 150 min and declined following the termination of A–HS. A significant (*p* ≤ 0.05) B by time interaction was observed such that the BETA group exhibited lower Tcore values than all other groups at the peak of A-HS (min. 150; Figure 1). Even 120 min after the A-HS has subsided (at 270 min), the CONT still had higher Tcore than all other groups (B by C by time interaction; *p* < 0.05). 

Changes in blood parameters in response to A–HS test are presented in Table 4. Packed cell volume (hematocrit %) were significantly altered by time (due to exposure to the A-HS; *p* < 0.05), such that—unlike the rise observed in the CONT group—values actually declined significantly in the BETA-treated groups (B by time interaction; *p* = 0.04). Furthermore, A-HS was responsible for significant declines (*p* < 0.05) in H:L ratio, an effect was also solely driven by C treatment (in CRET and COMB groups). No (*p* > 0.05) mentionable variations were noticed in other hemogramic parameters tested.

## 4. Discussion

### 4.1. Broiler Growth Performance

This study was conducted to evaluate the impact of dietary supplementation with B and/or C on heat-stressed broilers. With the exception of C-evoked alteration on FCR on the second week, B and/or C supplementation did not affect overall performance parameters (BW, FCR, FI, and BWG) investigated in this study. Our results are in agreement with the findings of Esteve-Garcia and Mack [27] and Pillai et al. [28], who supplemented broiler diet with B at different levels (0.05%, 0.05–0.10%, and 0.28%) without declaring any improvement on live performance. In addition, Zhang et al. [29] reported that live performance was not significantly affected when broiler birds were fed a C monohydrate dietary supplement. Moreover, Remus [30] reported that there were no clear differences in live performance due to adding B at 0.10% and 0.09% to the basal diet in turkeys. In contrast with the current study, Attia et al. [7], Hassan et al. [31], and Zhan et al. [8] reported an improvement in BWG and FCR of broilers among treatments supplemented with dietary B at different levels (0.04–0.07%, 0.07–0.14%, and 0.05%). In addition, C supplementation lead to enhanced FCR, breast muscle yield, and meat quality but reduced pH post-mortem and color brightness when compared with control broiler chicks [9]. Furthermore, C supplementation at 600 g/ton and blood meal at 5% in diet improved BWG compared to the control group [10]. A reasonable explanation of the increase in live performance for B-fed broiler birds was explained by Eklund et al. [32], being due to improving nutrient utilization and, as a result, increasing the availability of sulfur-containing amino acids (methionine and cysteine) for protein deposition in muscle. In the current study, it is to be mentioned that the brief improvement in FCR due to increased BWG noticed on the second week of the trial may be attributed to C supplementation (Table 2). In humans, bodybuilders in muscle-gaining phase recommend utilizing C as dietary supplements (3 g/day) to increase muscle mass [33]. In addition, C increases the bioavailability of phosphocreatine for cellular ATP production within the body, especially in active muscle. The supplementation of swine diets with C monohydrate increase antemortem muscle phosphocreatine levels, thereby sparing muscle glycogen. In this regard, James et al. [34] and Young et al. [14] reported that supplementing swine diets with C monohydrate reduced longissimus muscle drip losses. 

### 4.2. Breast Meat Quality and Color Values

In the current study, B and/or C supplementation did not significantly affect any of the examined meat-related parameters, except with the redness of breast muscle. However, Konca et al. [35] observed the effect of dietary B supplementation on quality parameters of broiler’ breast meat and reported no effect on breast meat pH value. In pigs, C saves phosphate inside the muscle cells, which leads to a higher buffer capacity in the cell, thereby slowing the post-mortem pH drop [36], which in turn decreases protein denaturation and losses of water in meat [37]. In contrast, Remus [30] stated a significant increase in breast muscle yield in broiler chicks. A rise in breast meat percentage under HS (35 °C for 10 h and reduced to 25 °C for the rest of the day) was also observed in B-treated chickens [38]. Furthermore, guanidinoacetic acid (GAA) supplementation at 1.2 g/kg of feed resulted in improved breast muscle yield with minor effect on meat quality, when compared to control broiler chick [9]. Unlike findings of Hur et al. [39], who found that meat redness was increased by B treatment in pigs, our current results show that redness declined. 

### 4.3. Thermoregulation and Hemogramic Profile

The HS is one of the major problems facing the poultry industry [2,40], especially in open system settings. During high environmental temperatures, chickens adapt by reducing FI to decrease endogenous heat production and hyperventilating or panting to eliminate extra heat. However, the results are detrimental to their productive pathways impeding growth rate and, hence, BW [41]. Additionally, such hindrances in performance parameters are often accentuated by nearly inevitable respiratory alkalosis [42]. Therefore, this study was designed to evaluate the impact of dietary supplementation of B and/or C on the performance and carcass characteristics of broilers under HS conditions. Interestingly, clear signs of HS mitigation were evident in the current trial. Hyperthermia was pronouncedly noticed in all treatment groups exposed to A-HS event. However, B treatment in particular managed to significantly alleviate this excess heat load (with primary reference to the CONT group). Only marginal effects of C or COMB treatments were found to improve chicks’ thermoregulatory performance under A-HS conditions.

Birds usually utilize panting as a mean of respiratory evaporative heat loss, with no chance of direct cutaneous evaporative heat loss (sweating). In light of our present findings, it is strongly suggested that such enhancements are mediated through water conservation mechanisms, as supported by the hematocrit responses. Packed cell volume rose overtime only in the CONT, and was yet found to decline in all other treatments. This proposition is also corroborated by visually observed behavioral signs seen at the mid A-HS episode, in which rehydration attempts were frequently noticed in the BETA, CRET, and COMB groups, while the CONT counterparts showed stronger sluggishness and, therefore, less rehydration (water drinking) momentum. In light of the improved thermoregulatory performance and water balance indicators, it seems that B and/or C have successfully improved water holding balance and therefore, helped in thermoregulatory performance. This also suggests that those birds managed to counterbalance evaporative water loss by drinking more and thus helped their survival. When chickens are exposed to HS, the content of lymphocytes in the blood decreases, the number of heterophils increases, and the H:L ratio increases [43]. Conversely, A-HS was responsible for significant declines in H:L ratio, an effect driven by C treatment in our study. These findings provide evidence that C supplementation may serve as an effective nutritional hyper-hydration strategy for chicks in hot environments thereby reducing risk of HS.

## 5. Conclusions 

Supplementation B enhanced birds’ resistance under thermophysiological conditions. Under the current study conditions, the results demonstrated that production parameters measured (BW, FCR, FI, and BWG) were not affected by B and/or C supplementation during the rearing period. In addition, carcass parameters and meat color were not affected by B and/or C supplementation, except the redness had higher value than in the control group. Moreover, the HS trial showed that B rendered the chicks more tolerant by alleviating their hyperthermic response with time. Furthermore, hematocrit and heterocytes/lymphocyte ratio in blood parameters were found to be affected by B and/or C supplementation, but the other tested parameters had no significant change. Further studies are needed to investigate the intra- and extracellular fluid dynamic changes driven by B and/or C.

## Figures and Tables

**Table 1:** Ingredients and chemical composition of basal experimental diets.

Ingredients (%)	Starter	Grower
Corn	61.00	61.60
Soybean Meal	34.20	32.20
Concentrate	2.50	2.50
Vegetable Oil	0.80	2.20
Limestone	1.20	1.20
Premixes	0.10	0.10
Antifungal	0.20	0.20
Total	100.0	100.0
Nutrients Composition (Calculated)		
Metabolizable energy, kcal/kg	3100	3150
Crude Protein (%)	22.50	21.50
Lysine (%)	1.46	1.28
Methionine (%)	0.52	0.49
Calcium	1.05	0.98
Available Phosphorus	0.51	0.46

**Table 2:** Means of body weight (BW), feed conversion ratio (FCR), feed intake (FI), and body weight gain (BWG) of broiler chicks ^1^ treated with dietary betaine (B) and/or creatine (C) ^2^.

Item	Age (Week)				
	1	2	3	4	5
BW (g/week)					
CONT (B−/C−)	173.40	469.00	909.80	1545.40	2195.80
BETA (B+/C−)	174.40	474.20	924.80	1512.20	2171.40
CRET (B−/C+)	176.30	490.40	934.60	1551.80	2228.00
COMB (B+/C+)	172.80	482.80	933.80	1544.00	2194.80
SEM ^3^	3.87	8.03	16.70	20.08	28.13
*p*-Value					
B	0.75	0.80	0.6	0.07	0.32
C	0.87	0.08	0.33	0.65	0.34
B × C	0.57	0.44	0.64	0.18	0.88
FCR (%)					
CONT (B−/C−)	1.40	1.30a	1.43	1.57	1.67
BETA (B+/C−)	1.37	1.27a	1.41	1.58	1.68
CRET (B−/C+)	1.32	1.22b	1.42	1.55	1.64
COMB (B+/C+)	1.40	1.22b	1.43	1.55	1.64
SEM ^3^	0.05	0.02	0.03	0.02	0.05
*p*-Value					
B	0.62	0.39	0.82	0.86	0.98
C	0.57	<0.01	0.90	0.25	0.51
B × C	0.27	0.29	0.55	0.72	0.96
FI (g/week)					
CONT (B−/C−)	185.71	384.99	630.53	996.61	1084.56
BETA (B+/C−)	183.71	380.80	635.01	925.45	1102.00
CRET (B−/C+)	179.65	380.86	628.71	957.50	1108.50
COMB (B+/C+)	184.82	377.45	643.19	945.08	1057.78
SEM ^3^	2.91	7.80	14.94	20.89	30.16
*p*-Value					
B	0.59	0.63	0.53	0.07	0.59
C	0.41	0.64	0.83	0.65	0.74
B × C	0.24	0.96	0.74	0.18	0.28
BWG (g/week)					
CONT (B−/C−)	133.40	295.60b	440.80	635.60	650.40
BETA (B+/C−)	134.40	299.80b	450.60	587.40	659.20
CRET (B−/C+)	136.30	314.10a	444.20	617.20	676.20
COMB (B+/C+)	132.80	310.00a	451.00	610.20	650.80
SEM ^3^	3.87	5.99	12.12	14.50	23.84
*p*-Value					
B	0.75	0.99	0.50	0.08	0.73
C	0.87	0.03	0.8	0.88	0.72
B × C	0.57	0.50	0.90	0.17	0.48

^1^ 440 broiler chicks (Indian River breed; age 35 days; *n* = 22/treatment). ^2^ Dietary treatments were 1 g B/kg and/or 1.2 g C/kg of feed. The control group (CONT) contained neither added B nor C. ^3^ Standard error of the means. BETA: Betaine group, CRET: Creatine group, COMB: Betaine and Creatine group.

**Table 3:** Means of carcass dressing (%), pH, breast meat cooking loss (%), water holding capacity (WHC, %), shear force (SF) Kg/cm^2^, and breast meat color values of broiler chicks fed dietary betaine (B) and/or creatine (C).

Variable ^1^	Treatment Groups ^2^				SEM ^3^	*p*-Value
	CONT	BETA	CRET	COMB		
Carcass dressing (%)	76.08	75.15	77.71	76.89	1.03	0.268
pH	6.03	6.03	6.03	6.08	0.02	0.267
Cooking loss (%)	26.02	26.11	26.11	27.97	0.95	0.415
WHC (%)	33.22	31.70	34.03	33.32	1.64	0.793
SF (Kg/cm^2^)	4.15	4.54	4.68	4.97	0.48	0.677
Lightness	46.37	45.86	45.67	47.17	6.0	0.330
Redness	31.57a	26.74bc	24.46c	31.27ab	1.6	0.011
Yellowness	19.36	19.33	19.65	19.38	2.4	0.805

Means within the same row with varying superscripts differ significantly (betaine by creatine interaction). ^1^ Parameters are based upon breast meat samples taken on day 35 (slaughter day; 10 chicks/treatment group). ^2^ Dietary treatments were 1 g B/kg and/or 1.2 g C/kg of feed. The control group (CONT) contained neither added B nor C for a total 35 days. ^3^ Standard error of the mean. BETA: Betaine group, CRET: Creatine group, COMB: Betaine and Creatine group.

**Table 4:** Means of blood parameters in broiler chicks ^1^ treated with dietary betaine (B) and/or creatine (C) and experiencing acute heat stress.

Parameter	Treatment Group ^2^					Effect-Associated *p*-Value ^4^			
	CONT	BETA	CRET	COMB	SEM ^3^	B	C	B × C	Time
White blood cells (×103/µL)						NS	NS	NS	NS
Pre–heat stress	10.88	9.11	9.23	5.75	1.43				
Post–heat stress	6.69	9.73	8.67	7.20	1.27				
Red blood cells (×106/µL)						NS	NS	NS	NS
Pre–heat stress	2.57	2.49	2.60	2.83	0.31				
Post–heat stress	2.34	2.91	2.41	2.75	0.22				
Hemoglobin (g/dL)						NS	NS	NS	NS
Pre–heat stress	10.88	11.45	11.71	11.98	0.57				
Post–heat stress	11.39	10.27	10.73	10.38	0.50				
Hematocrit (%)						NS	NS	NS	**
Pre–heat stress	31.96	31.50	31.63	33.17	1.82				♦
Post–heat stress	32.30	28.00	28.63	28.67	1.61				
(MCV; fL)						NS	NS	NS	NS
Pre–heat stress	127.92	125.18	123.91	121.90	12.72				
Post–heat stress	143.41	106.25	119.47	107.70	9.00				
(MCH; pg)						NS	NS	NS	NS
Pre–heat stress	42.81	45.42	45.84	44.22	4.11				♦
Post–heat stress	50.51	38.94	44.98	39.27	3.63				
(MCHC; g/dL)						NS	NS	NS	NS
Pre–heat stress	34.26	36.32	37.05	36.10	1.44				
Post–heat stress	35.43	36.79	37.58	36.45	1.28				
Thrombocytes (×103/µL)						NS	NS	NS	NS
Pre–heat stress	26.04	24.36	21.40	22.67	4.62				
Post–heat stress	23.09	23.28	18.97	22.67	4.08				
Heterocytes (H; %)						NS	*	NS	NS
Pre–heat stress	50.00	54.10	60.75	61.50	4.45				
Post–heat stress	50.00	51.60	52.18	51.00	3.93				
Lymphocytes (L; %)						NS	*	NS	*
Pre–heat stress	42.56	38.00	32.50	33.17	4.63				
Post–heat stress	43.56	42.00	41.07	41.67	4.09				
Monocytes (%)						NS	NS	NS	NS
Pre–heat stress	5.89	6.30	6.00	5.17	0.37				
Post–heat stress	5.89	5.80	6.00	6.67	0.38				
H:L ratio						NS	*	NS	*
Pre–heat stress	1.23	1.59	2.03	1.90	0.36				
Post–heat stress	1.20	1.18	1.45	1.28	0.32				

^1^ Broiler chicks (age 31 days; *n* = 20) were exposed to 150 min of heat stress (Ta = 34.45 ± 0.20 °C; RH = 37.60 ± 0.28%), after which thermoneutrality was resumed (Ta = 25.77 ± 0.28 °C; RH = 32.28 ± 0.40%). (*n* = 10 chicks/treatment). All chicks were transported from floor open system farming 48 h prior to the heat challenge test for new-housing adaptation. ^2^ Dietary treatments were 1 g betaine (B)/kg and/or 1.2 g creatine (C)/kg of feed. The control group (CONT) contained neither added B nor C. ^3^ Standard error of the mean. MCV: Mean Corpuscular Volume; MCH: Mean Corpuscular Hemoglobin; MCHC: Mean Corpuscular Hemoglobin Concentration; BETA: Betaine group; CRET: Creatine group; COMB: Betaine and Creatine group. ^4^ NS = not significant (*p* > 0.05); * = significant (*p* ≤ 0.05); ** = significant (*p* < 0.01); ♦ = significant B by time interaction (*p* ≤ 0.05).

## References

[1] Hossain M.A., Suvo K.B., Islam M.M. (2011). Performance and economic suitability of three fast–growing broiler strains raised under farming condition in Bangladesh. Int. J. Agric. Res. Innov. Technol..

[2] Al-Dawood A., Büscher W. (2014). Air velocity produced by different types of mixing and ceiling fans to reduce heat stress in poultry houses. Int. J. Agric. For..

[3] Al-Dawood A. (2016). Applications of acute phase proteins as biomarkers in poultry. Bull. Fac. Agric. Cairo Univ..

[4] Al-Ruwaili M., Herzallah S., Al-Dmoor H., Al-Atiyat R. (2014). Effect of broiler commercial strains on total and free cholesterol levels of chicken muscle tissues. Glob. Vet..

[5] Husna A., Badruzzaman A.T.M., Runa N.Y., Yesmin S., Runa N.S., Rahman M.A., Mia M.M. (2017). Evaluation of productive performance of selected broiler strains under field condition at Sylhet district of Bangladesh. Ann. Vet. Anim. Sci..

[6] Department of Statistics, Amman Agricultural Surveys, Jordan (2016). Jordan Statistical Yearbook.

[7] Attia Y., Hassan R.A., Shehatta M.H., Abd El-Hady S.B. (2005). Growth, carcass quality and serum constituents of slow growing chicks as affected by betaine addition to diets containing 2. Different levels of methionine. Int. J. Poult. Sci..

[8] Zhan X.A., Li J.X., Xu Z.R., Zhao R.Q. (2006). Effects of methionine and betaine supplementation on growth performance, carcass composition and metabolism of lipids in male broilers. Br. Poult. Sci..

[9] Michiels J., Maertens L., Buyse J., Lemme A., Rademacher M., Dierick N.A., de Smet S. (2012). Supplementation of guanidinoacetic acid to broiler diets: Effects on performance, carcass characteristics, meat quality and energy metabolism. Poult. Sci..

[10] Carvalho C.M.C., Fernandes E.A., de Carvalho A.P., Maciel M.P., Caires R.M., Fagundes N.S. (2013). Effect of creatine addition in feeds containing animal meals on the performance and carcass yield of broilers. Rev. Bras. Ciência Avícola.

[11] Cholewa J.M., Guimaraes-Ferreira L., Zanchi N.E. (2014). Effects of betaine on performance and body composition: A review of recent findings and potential mechanisms. Amino Acids.

[12] Hruby M., Ombabi A., Schlagheck A. Natural betaine maintains layer performance in methionine/choline chloride reduced diets. Proceedings of the 15th European Symposium on Poultry Nutrition.

[13] Eklund M., Mosenthin R., Piepho H.P. (2006). Effects of betaine and condensed molasses solubles on ileal and total tract nutrient digestibilities in piglets. Acta Agric. Scand Sect. A Anim. Sci..

[14] Young J.F., Bertram H.C., Rosenvold K., Lindahl G., Oksbjerg N. (2005). Dietary creatine monohydrate affects quality attributes of Duroc but not Landrace pork. Meat Sci..

[15] Hultman E., Soderlund K., Timmons J.A., Cederblad G., Greenhaff P.L. (1996). Muscle creatine loading in men. J. Appl. Physiol..

[16] Lara L.J., Rostagno M.H. (2013). Impact of heat stress on poultry production. Animals.

[17] Al-Dawood A. (2015). Adoption of agricultural innovations: Investigating current status and barriers to adoption of heat stress management in small ruminants in Jordan. Am.-Eurasian J. Agric. Environ. Sci..

[18] Silanikove N., Koluman N. (2015). Impact of climate change on the dairy industry in temperate zones: Predications on the overall negative impact and on the positive role of dairy goats in adaptation to earth warming. Small Rumin. Res..

[19] Virden W.S., Kidd M.T. (2009). Physiological stress in broilers: Ramifications on nutrient digestibility and responses. J. Appl. Poult. Res..

[20] Rezaei M., Moghaddam H.N., Reza J.P., Kermanshahi H. (2004). The effect of dietary protein and lysine levels on broiler performance and carcass characteristics and N excretion. Int. J. Poult. Sci..

[21] Hudspeth J., Lyon C.E., Lyon B.G., Mercuri A.J. (1973). Weights of broiler parts as related to carcass weights and type of cut. J. Food Sci..

[22] Abdullah A., Matarneh S. (2010). Broiler performance and the effects of carcass weight, broiler sex and postchill carcass aging duration on breast fillet quality characteristics. J. Appl. Poult. Res..

[23] Sams A., Janky D. (1986). The influence of brine chilling on tenderness of hot-boned, chill-boned and age-boned broiler breast fillets. Poult. Sci..

[24] Horcada A., Beriain M.J., Purroy A., Lizaso G., Chasco J. (1998). Effect of sex on meat quality of Spanish lamb breeds (Lacha and Rasa Aragonesa). Anim. Sci..

[25] SAS Institute Inc. (2012). SAS/STAT® 12.1 User’s Guide.

[26] Littell R.C., Milliken G.A., Stroup W.W., Wolfinger R.D., Schabenberger O. (2006). SAS for Mixed Models.

[27] Esteve-Garcia E., Mack S. (2000). The effect of DL-methionine and betaine on growth performance and carcass characteristics in broilers. Anim. Feed Sci. Technol..

[28] Pillai P.B., Fanatico A.C., Beers K.W., Blair M.E., Emmert J.L. (2006). Homocysteine remethylation in young broilers fed varying levels of methionine, choline and betaine. Poult. Sci..

[29] Zhang L., Li J.L., Gao T., Lin M., Wang X.F., Zhu X.D., Gao F., Zhou G.H. (2014). Effects of dietary supplementation with creatine monohydrate during the finishing period on growth performance, carcass traits, meat quality and muscle glycolytic potential of broilers subjected to transport stress. Animals.

[30] Remus J. (2001). Betaine for increased breast meat yield in turkeys. World Poult..

[31] Hassan R.A., Attia Y.A., El-Ganzory E.H. (2005). Growth, carcass quality and serum constituents of slow growing chicks as affected by betaine addition to diets containing 1. Different levels of choline. Int. J. Poult. Sci..

[32] Eklund M., Bauer E., Wamatu J., Mosenthin R. (2005). Potential nutritional and physiological functions of betaine in livestock. Nutr. Res. Rev..

[33] Iraki J., Fitschen P., Espinar S., Helms E. (2019). Nutrition recommendations for bodybuilders in the off-season: A narrative review. Sports.

[34] James B.W., Goodband R.D., Unruh J.A., Tokach M.D., Nelssen J.L., Dritz S.S., O’Quinn P.R., Andrews B.S. (2002). Effect of creatine monohydrate on finishing pig growth performance, carcass characteristics and meat quality. Anim. Feed Sci. Technol..

[35] Konca Y., Kirkpinar F., Mert S., Yaylak E. (2008). Effects of betaine on performance, carcass, bone and blood characteristics of broilers during natural summer temperatures. J. Anim. Vet. Adv..

[36] Cromwell G.L., Lindemann M.D., Randolph J.R., Monegue H.J., Laurent K.M., Parker G.R. (1999). Efficacy of betaine as a carcass modifier in finishing pigs fed normal and reduced energy diets. J. Anim. Sci..

[37] Matthews J.O., Southern L.L., Higbie A.D., Persica M.A., Bidner T.D. (2001). Effects of betaine on growth, carcass characteristics, pork quality and plasma metabolites of finishing pigs. J. Anim. Sci..

[38] Enting H., Eissen J., de los Mozos J., del Álamo A.G., Ayala P.P. TNI betaine improves broiler chicken performance and carcass quality under heat stress conditions. Proceedings of the 16th European Symposium on Poultry Nutrition.

[39] Hur S.J., Yang H.S., Park B., Joo S.T. (2007). Effects of dietary glycine betaine on pork quality in different muscle types. Asian Aust. J. Anim. Sci..

[40] St-Pierre N.R., Cobanov B., Schnitkey G. (2003). Economic losses from heat stress by US livestock industries. J. Dairy Sci..

[41] Quinteiro-Filho W.M., Ribeiro A., Ferraz-de-Paula V., Pinheiro M.L., Sakai M., Sá L.R., Ferreira A.J., Palermo-Neto J. (2010). Heat stress impairs performance parameters, induces intestinal injury and decreases macrophage activity in broiler chickens. Poult. Sci..

[42] Borges S.A., Fischer da Silva A.V., Majorka A., Hooge D.M., Cummings K.R. (2004). Physiological responses of broiler chickens to heat stress and dietary electrolyte balance (sodium plus potassium minus chloride, milliequivalents per kilogram). Poult. Sci..

[43] Aengwanich W., Suttajit M. (2010). Effect of polyphenols extracted from Tamarind (*Tamarindus indica* L.) seed coat on physiological changes, heterophil/lymphocyte ratio, oxidative stress and body weight of broilers (*Gallus domesticus*) under chronic heat stress. Anim. Sci. J..

